# Optimized Probe Masking for Comparative Transcriptomics of Closely Related Species

**DOI:** 10.1371/journal.pone.0078497

**Published:** 2013-11-08

**Authors:** Yvonne Poeschl, Carolin Delker, Jana Trenner, Kristian Karsten Ullrich, Marcel Quint, Ivo Grosse

**Affiliations:** 1 Martin Luther University Halle–Wittenberg, Institute of Computer Science, Halle (Saale), Germany; 2 Leibniz Institute of Plant Biochemistry, Department of Molecular Signal Processing, Halle (Saale), Germany; 3 German Center of Integrative Biodiversity Research (iDiv) Halle-Jena-Leipzig, Leipzig, Germany; Queen’s University Belfast, United Kingdom

## Abstract

Microarrays are commonly applied to study the transcriptome of specific species. However, many available microarrays are restricted to model organisms, and the design of custom microarrays for other species is often not feasible. Hence, transcriptomics approaches of non-model organisms as well as comparative transcriptomics studies among two or more species often make use of cost-intensive RNAseq studies or, alternatively, by hybridizing transcripts of a query species to a microarray of a closely related species. When analyzing these cross-species microarray expression data, differences in the transcriptome of the query species can cause problems, such as the following: (i) lower hybridization accuracy of probes due to mismatches or deletions, (ii) probes binding multiple transcripts of different genes, and (iii) probes binding transcripts of non-orthologous genes. So far, methods for (i) exist, but these neglect (ii) and (iii). Here, we propose an approach for comparative transcriptomics addressing problems (i) to (iii), which retains only transcript-specific probes binding transcripts of orthologous genes. We apply this approach to an *Arabidopsis lyrata* expression data set measured on a microarray designed for *Arabidopsis thaliana*, and compare it to two alternative approaches, a *sequence-based* approach and a genomic DNA *hybridization-based* approach. We investigate the number of retained probe sets, and we validate the resulting expression responses by qRT-PCR. We find that the proposed approach combines the benefit of sequence-based stringency and accuracy while allowing the expression analysis of much more genes than the alternative sequence-based approach. As an added benefit, the proposed approach requires probes to detect transcripts of orthologous genes only, which provides a superior base for biological interpretation of the measured expression responses.

## Introduction

While RNAseq approaches gained increased popularity for transcriptome analyses, microarrays are still in use due to their simplicity and easier data processing. In addition, a plethora of tools, comparable data sets, and experiences make microarrays attractive, also in transcriptomics studies of non-model organisms or in comparative transcriptomics studies among different species. However, one problem in cross-species analyses is that microarrays are usually designed for a specific *reference species*. If no specific microarray is available for a *query species*, the microarray of a closely related species can be utilized, as sequences tend to be more similar among closely related species.

Sequence differences in target genes of the query species can cause three problems. First (i), the hybridization signal can be reduced due to mismatches or deletion of the target. Second (ii), the hybridization signal can be increased due to cross hybridization, where probes do not only detect the transcript of the intended target gene but hybridize also to transcripts of other genes [1]–[3]. And third (iii), probes can target transcripts that are highly similar in the targeted region, but are not products of orthologous genes in the reference and the query species.

Two popular approaches addressing problem (i) are the *sequence-based* approach by [4] and the genomic DNA *hybridization-based* approach by [5]–[7]. The sequence-based approach by [4] uses the transcript of the annotated target gene of the reference species to determine the transcript of the target gene of the query species prior. Afterwards, the sequences of the target transcripts of the query species are compared to the probe sequences of the microarray of the reference species to identify and mask probes that are affected by the mentioned problem (i). The sequence-based approach retains probes that perfectly match the sequences of the target transcripts of the query species.

The hybridization-based approach by [5]–[7] hybridizes genomic DNA (gDNA) of the query species to the microarray of the reference species to detect probes affected by problem (i). The hybridization-based approach by [7] address problem (i) by masking probes below a given gDNA hybridization intensity value. However, it retains probes that possibly match regions on the genomic DNA outside transcribed regions.

Common to both approaches is that they fail to provide solutions for problem (ii) and (iii), the problems of cross hybridization and transcripts of non-orthologous genes, respectively.

Here, we propose a sequence-based approach similar to [4], but in contrast to [4], we account for a slight sequence divergence of the query species to the reference species by allowing probes to match the target transcript with at most one mismatch. We additionally address problems (ii) and (iii) to facilitate reliable comparative transcriptomics analyses.

We apply the proposed approach (*1* *mm* ) to an auxin expression data set of *A. lyrata* measured on the Affymetrix ATH1-121501 microarray designed for *A. thaliana* [8] and compare it to the sequence-based approach (*0* *mm* ) by [4], the gDNA hybridization-based approach (*gDNA* ) by [7], and a *naive* approach that uses all probes on the microarray.

We investigate the effect of using probes matching with a single mismatch in contrast to using only perfectly matching probes, and we additionally study the effect of addressing problems (ii) and (iii). Therefore, we compare the number of transcript-specific probe sets retained by each of the three masking approaches and the naive approach. We validate the accuracy of the resulting expression responses for the auxin-treated query species *A. lyrata* by qRT-PCR.

## Methods

### 1 mm Approach

To facilitate a reliable comparative transcriptomics analysis based on microarray hybridization of a reference species and a closely related query species, transcript-specific probes must be separated from probes affected by at least one of the problems (i) to (iii) mentioned in the introduction. We declare a probe to be transcript-unspecific if it is affected by cross hybridization or if it targets transcripts of non-orthologous genes. Our goal is to detect and mask transcript-unspecific probes and probes that target no transcript from subsequent analyses.

**Figure 1:**
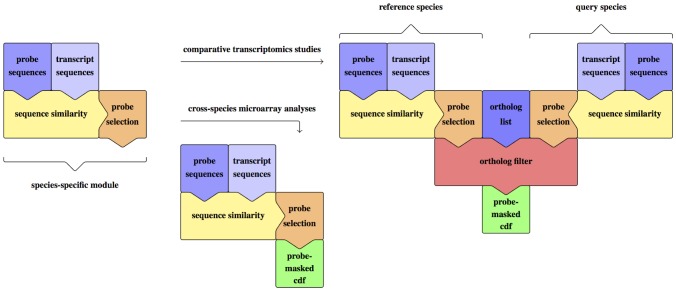
The two possible workflows of the *1* *mm* approach. The *1* *mm* approach can be used in two different ways: For cross-species microarray analyses or for comparative transcriptomics studies. Each of the two workflows results in a probe-masked cdf colored in green. The tips of the colored pieces show the flow of information. The blue colored pieces show the input data provided by the user, whereas the yellow, orange, and red pieces show the two or three steps of the *1* *mm* approach leading to a probe mask. The *species-specific module* consists of the sequence similarity step with the microarray-specific probe sequences and the species-specific transcript sequences as input, and the probe selection step that results in a list of probe sets containing only reliable probes. The *species-specific module* can be used for generating a probe-masked cdf for cross-species microarray analyses of non-model species. Two different *species-specific modules* can be used with an orthologous gene list for generating a probe-masked cdf for comparative transcriptomics studies.

We generate a probe mask for comparative transcriptomics in four steps (Figure 1): first, we align the sequences of the probes to all known transcripts, including all known *isoforms*, of the reference and the query species to find regions of high similarity. Here and in the following, the term *isoform* refers to *one possible transcript of a gene*. Second, we filter the alignments and mask probes that match no transcripts or are affected by cross-hybridization. Third, we verify if the transcripts targeted by a probe set are transcripts of orthologous genes of the reference and the query species. Fourth, we generate the final probe mask based on the outcome of the previous three steps.

#### Sequence similarity

To find regions of high similarity, we use *BLASTN-short* of the BLAST+ package [9] for computing an alignment of the sequences of the perfectly matching probes present on the Affymetrix microarray of the reference species to the protein coding transcripts of the reference and query species, respectively. We require BLASTN-short to align all 25 bases of the probe sequences and allow at most one mismatch (perc_identity = 96 and ungapped = 1) to account for sequence variation in the transcriptome of the query species. We record for each probe all matching transcripts. Subsequently, we process the results of the alignment for the reference species and the query species separately but follow the same *probe selection* steps.

#### Probe selection

**Figure 2:**
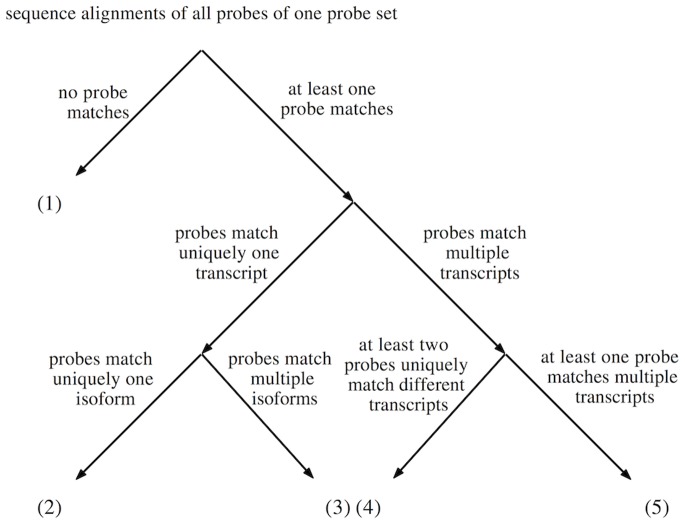
Assignment of a probe set to one of five groups. The assignment of a probe set to a specific group depends on the characteristics of the matching probes in the probe sets. The term *isoform* refers to one possible transcript of a gene.

We subdivide the probe sets into five disjoint groups (Figure 2) consisting of probe sets in which: (1) none of the probes match any transcript, (2) all matching probes uniquely match one isoform of one gene, (3) all matching probes uniquely match several isoforms of one gene, (4) at least two of the matching probes uniquely match different transcripts, and (5) at least one of the matching probes matches multiple transcripts. We process the probe sets of the five groups as follows:

1. Mask all probes.
2. Mask probes that do not match.
3. Mask probes that do not match.
4. Mask probes that do not match any transcript.
5. Mask probes that do not match any transcript and mask probes that match multiple transcripts.

We mask probes in probe sets of groups (1) to (5) matching no transcript because they are expected to generate no gene-specific hybridization signals. We compute for each probe set of group (3) the union of probes matching any known isoform of a specific gene. Probe sets of group (4) contain probes that uniquely match different transcripts. For each matching transcript, we process the respective probes according to the rules of groups (2) or (3). Probe sets of group (5) contain at least one probe that matches multiple transcripts and thus is affected by cross hybridization. We mask such probes and process the remaining probes according to the rules of the four previous groups.

As a result of the masking, the number of unmasked probes within a probe set can vary from zero to 11. We mask probe sets containing less than three probes, because we consider these unreliable [10]. We perform the processing step for the alignment of the reference species and the query species, which results in two species-specific modules, which return two lists of *mappings* of probe sets to genes, one for the reference and one for the query species, respectively (Figure 1).

#### Filtering of orthologous genes

We join both lists of mappings by the probe set names to obtain gene-pair-matchings of the corresponding probe sets. Multiple gene pairs are possible for probe sets belonging to groups (4) or (5), because they can target multiple transcripts. We consider a gene pair orthologous if it is contained in the list of orthologs (Methods List of orthologous genes) of the reference species and the query species.

If a gene pair is orthologous, we compute the intersection of the probes. We mask probes that are not contained in the intersection and thus do not target both transcripts. For probe sets of groups (4) and (5) we keep the orthologous gene pair with the largest number of probes. We again mask a probe set if it contains less than three probes after the intersection.

This filtering step leads to a *final list* of probe sets targeting only transcripts of orthologous genes using the same probes.

#### Probe masking

We modify the original chip definition file (cdf), which contains the locations of the probes on the respective microarray, based on the final list of probe sets. We create the probe-masked cdf using the function make.cdf.package of the makecdfenv package [11] to allow using the probe-masked cdf with R [12] for further analyses. The probe-masked cdf can be used by the function read.affybatch of the affy package [13].

### Data Sets

#### Transcript sequences

We obtain the transcript data sets of *A. thaliana* [14] and *A. lyrata* [15] from Phytozome v7.0 (http://www.phytozome.com). These data sets contain sequences of transcripts of 35386 and 32670 protein-coding genes for *A. thaliana* and *A. lyrata*, respectively.

#### Probe sequences

We obtain the sequences of the probes of the ATH1-121501 microarray from Affymetrix (http://www.affymetrix.com). The data set comprises 251078 sequences including 975 sequences of control probes.

#### Target sequences

We obtain the sequences of the targets of the ATH1-121501 microarray from Affymetrix (http://www.affymetrix.com) and proceed according to [4]. The data set comprises 22814 sequences.

#### List of orthologous genes

We obtain the protein sequences of *A. thaliana* and *A. lyrata* from Phytozome v7.0 (http://www.phytozome.com). These data sets contain protein sequences of 35386 and 32670 protein-coding genes for *A. thaliana* and *A. lyrata*, respectively. We generate the list containing orthologous genes of *A. thaliana* and *A. lyrata* using BLASTP [16] setting the maximal E-value to 1e-05 and retaining only the best BLASTP hit.

#### gDNA hybridization data set

We obtain the.cel file containing the hybridization intensities of the gDNA of *A. lyrata* from http://affy.arabidopsis.info/xspecies/and proceed it according to [7].

#### Chip definition file

We obtain the chip definition file (cdf) for the ATH1-121501 microarray from Affymetrix (http://www.affymetrix.com). The cdf contains the locations of the PM and the MM probes of the ATH1-121501 microarray which target the 3′-end of *A. thaliana* transcripts.

#### Expression data set

We obtain a cross-species hybridization data set of *A. thaliana* and *A. lyrata* using the ATH1 microarray from NASC (http://affymetrix.arabidopsis.info/narrays/experimentpage.pl?experimentid=579). The data set assesses the variation of auxin responses in seven days old *A. thaliana* and *A. lyrata* seedlings. Information on the experimental procedures are provided at http://affymetrix.arabidopsis.info/narrays/experimentpage.pl?experimentid=579.

We load the.cel files into R [12] using the masked cdfs resulting from the 1 mm approach (Methods 1 mm approach), the 0 mm approach, the gDNA approach, and the non-masked cdf of the naive approach, and the affy package. We perform background correction, quantile normalization, and summary of the expression data using RMA [17] of the simpleaffy package [18], which returns 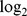-transformed expression values.

### qRT-PCR Analysis

We perform a verification of transcription levels by qRT-PCR, to assess the accuracy of the expression responses resulting from the four studied approaches. Plant material was subjected to the same experimental conditions as described in Methods. 3 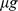 of total RNA was subjected to reverse transcription using the RevertAid First Strand cDNA Synthesis Kit by Fermentas according to the manufacturers description. Power SYBR Green PCR Master Mix (Applied Biosystems) was used for subsequent quantitative real-time PCR analyses. Expression of the PP2A catalytic subunit gene AT1G13320 (array element: 259407_at) served as the constitutively expressed reference gene [19]. Comparative expression levels (CELs) for the respective genes of interest were calculated as 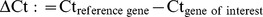. Oligonucleotide sequences and a complete list of analyzed genes are presented in Supplementary Table S3.

### Candidate Selection

We choose 40 genes as candidate genes for verification by qRT-PCR based on the response to auxin treatment and the composition of the corresponding probe sets (Table 1 and Supplementary Table S4). The number of probes per probe set ranges from three to ten, and the number of imperfectly matching probes with a single mismatch ranges from zero to all. We choose these 40 candidate genes from four categories:

**Table 1:** A table of verified candidate genes.

ae name	locus At	locus Al	probes 1 mm	probes 0 mm	probes gDNA	ΔΔCt	*1 mm	*0 mm	*gDNA	*naive	category
245245_at	AT1G44318	314128	110-10-0	-0-0-0	11-xxxx-0-	−1.64	−1.46	−2.04	−0.22	−0.35	A,B,C,D
245696_at	AT5G04190	939816	0-0-1-001-	0-0-00-	0x0x-00-x-	3.03	1.68	2.53	0.98	0.55	A,B,C,D
246270_at	AT4G36500	490986	10110-110	-0-0-0	1-0-x-11-	−2.27	−2.18	−2.80	−1.42	−1.69	A,B,C,D
248676_at	AT5G48850	494948	00-11001-10	00-00-0	-0-1-001x10	3.30	1.86	2.77	2.07	1.55	A,B,C,D
251705_at	AT3G56400	486080	00-10-1-0	00-0-0	0-x10-1-x-	−2.90	−2.46	−2.90	−1.81	−1.36	A,B,C,D
252205_at	AT3G50350	485386	0-0-0-0-1	0-0-0-0-	0x-x0x0x0x1	1.82	1.36	1.65	0.31	0.47	A,B,C,D
252626_at	AT3G44940	484892	-11010-0-	-0-0-0-	-10-0-0-	−1.14	−0.85	−0.93	−1.22	−0.42	A,B,C,D
253287_at	AT4G34270	491240	01100001-00	0-0000-00	0-100-01-00	−0.10	−0.05	−0.10	−0.07	−0.08	A,B,C,D
253908_at	AT4G27260	492072	101-0-011	-0-0-0-	101x0xx-011	3.11	2.73	2.70	2.46	2.42	A,B,C,D
254175_at	AT4G24050	492457	-000100-	-000-00-	-x-00-00xx	−1.14	−1.01	−0.95	−0.60	−0.64	A,B,C,D
255788_at	AT2G33310	482270	1101110-00-	-0-0-00-	11011-0-00x	2.46	2.09	2.22	2.01	1.94	A,B,C,D
256131_at	AT1G13600	920239	1-101-0100	-0-0-00	1-1-1-0-00	−1.21	−0.83	−0.82	−1.03	−0.61	A,B,C,D
257153_at	AT3G27220	936451	11-00010-	-000-0-	-1xx-00-0-x	−3.95	−3.80	−4.21	−3.65	−3.24	A,B,C,D
259407_at	AT1G13320	920212	-000000-000	-000000-000	-000000-000	−0.35	0.10	0.09	0.10	0.10	A,B,C,D
260904_at	AT1G02450	470205	-0-0011110	-0-00-0	x-xx-0111-	−2.13	−1.29	−1.05	−0.83	−0.61	A,B,C,D
261892_at	AT1G80840	477161	-0000-1100	-0000-00	x-0000x-0-	−2.40	−2.27	−2.55	−2.05	−1.56	A,B,C,D
263970_at	AT2G42850	346095	0100001-1-1	0-0000-	0-00-01-1	−1.67	−1.11	−0.82	−1.16	−0.97	A,B,C,D
264867_at	AT1G24150	313260	010001-1-0-	0-000-0-	-1-01-0x	−1.37	−1.63	−1.85	−0.92	−1.27	A,B,C,D
265452_at	AT2G46510	483808	001-00-	00-x-00-	00-0-xx-	−1.89	−1.43	−1.35	−0.26	−0.20	A,B,C,D
265856_at	AT2G42430	935111	000-010-10	000-0-0-0	-00xx0-0-10	1.98	1.65	2.03	0.99	0.97	A,B,C,D
245336_at	AT4G16515	493225	-111-1-11	–	-1-1-x1xx11	2.76	2.16	NA	0.97	0.70	C,D
245369_at	AT4G15975	329916	-0-1-1-	–	-xx-x-	−2.48	−1.64	NA	−0.13	−0.15	C,D
245397_at	AT4G14560	946923	-1-110-	–	x1-x-x11-x-	2.74	2.37	NA	1.44	1.36	C,D
246993_at	AT5G67450	496850	0-01-1	–	0-x-0-xx-	−2.57	−0.80	NA	−1.10	−0.29	C,D
247524_at	AT5G61440	496303	-01-01-1	–	-01-01xx1	−3.48	−2.49	NA	−1.22	−0.77	C,D
248858_at	AT5G46630	948276	1-010-	–	1-010xx-xx	−0.10	0.28	NA	0.25	0.14	C,D
250937_at	AT5G03230	939701	10-110-1	–	10-1-0xxx1	−2.64	−2.74	NA	−2.05	−1.94	C,D
251910_at	AT3G53810	485775	-1-0-0-	–	x1xxx-xx0-	−1.31	−1.10	NA	−0.28	−0.24	C,D
253400_at	AT4G32860	491410	-11-010-1	–	x-11x0-0xx1	−1.42	−0.91	NA	−0.09	−0.12	C,D
253959_at	AT4G26410	945436	1011-01-	–	-11-xxx0-	−0.27	0.07	NA	0.08	0.00	C,D
261766_at	AT1G15580	471758	-1-0-111-	–	x-1xx0x-x	4.46	1.54	NA	0.38	0.61	C,D
262085_at	AT1G56060	474673	-1–100	–	xxx-x-xx100	1.70	1.67	NA	0.38	0.15	C,D
265256_at	AT2G28390	481666	-1-11100	–	-xx-11-0-	0.10	0.11	NA	−0.01	0.14	C,D
266649_at	AT2G25810	932757	11-01-1-	–	11-1-x-x	−0.68	−0.44	NA	−1.05	−0.72	C,D
266820_at	AT2G44940	483623	1-0-111-11-	–	-0-11-x-1-	−2.24	−1.44	NA	−1.73	−0.87	C,D
266974_at	AT2G39370	482956	-11-1-1011	–	-1-x-1011	4.02	0.85	NA	1.56	0.67	C,D
254761_at	AT4G13195	333009	-0-1-0	–	–	2.22	1.70	NA	NA	0.33	D
265806_at	AT2G18010	931672	1111-100-	–	–	3.96	0.62	NA	NA	0.53	D
247215_at	AT5G64905	951330	-000-	-000-	–	−4.60	−1.98	−1.85	NA	−0.03	B,D
248539_at	AT5G50130	495070	-01-00-	-0-00-	–	2.03	1.03	1.61	NA	0.19	B,D

* : 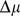, ae: array element, At: *Arabidopsis thaliana*, Al: *Arabidopsis lyrata*.

For each gene the corresponding array element name and the orthologous gene pair (locus *A. thaliana* by the TAIR id and locus *A. lyrata* by the Phytozome gene id) are listed. Additionally, the composition of the probe set in the 1 mm mask, the 0 mm mask, and the gDNA mask are shown. Originally, each probe set consists of 11 probes. The “–” represents a masked probe, “0” a perfectly matching probe, “1” a probe matching with one mismatch, and “x” represents a transcript-unspecific probe. The 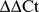 labeled column contains the 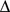 expression values of the 1 h treatment versus no treatment experiments derived from qRT-PCR. The next four columns contain the 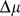 expression values of the 1 h treatment versus no treatment experiments derived from the three probe masking approaches and the non-masking approach. The last column contains the category used for computation of the Pearson correlation coefficient.

(A): 20 candidate genes present in all four approaches.
(B): 22 candidate genes: 20 genes of category (A) and 2 candidate genes present in the naive, 0 mm, and 1 mm approaches.
(C): 36 candidate genes: 20 genes of category (A) and 16 candidate genes present in the naive, gDNA, and 1 mm approaches.
(D): 40 candidate genes: 20 genes of category (A) and 20 candidate genes present in the naive and 1 mm approaches.

We choose genes of categories (A) and (B) for studying the impact of using probes with a single mismatch and removing probes affected by cross hybridization. We use genes of category (C) for studying the effect of using probes with a single mismatch and removing probes affected by cross hybridization on a larger set of genes, which contains 16 genes that are not retained by the 0 mm approach. We use genes of category (D) for studying the overall performance of the 1 mm approach.

### Correlation Analysis

We compute the mean 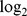 expression values of the 1 hour post auxin treatment samples and control samples (n = 3 biological replicates) for each of the 40 candidate genes, for the 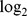 expression values resulting from the three masking approaches and the non-masking approach. We compute the log-fold changes (responses), which are the differences of the mean 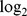 expression values of treated (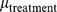) and control (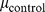) samples as 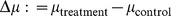. Similarly, we calculate the 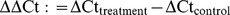 values of the comparative expression levels produced by qRT-PCR (Methods qRT-PCR analysis) of all candidate genes. We compute Pearson, Spearman, and Kendall correlation coefficients for all four candidate gene categories between the log-fold changes 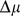 resulting from each approach and the 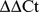 values resulting from qRT-PCR.

### Source Code

Source code is available at http://sourceforge.net/projects/probemaskingpipeline online.

## Results and Discussion

For a reliable comparative transcriptomics analysis of a reference species and a closely related query species based on microarray hybridization, transcript-specific probes must be separated from (i) probes matching no transcripts in at least one of the species, and transcript-unspecific probes that (ii) are affected by cross hybridization when they target multiple transcripts or (iii) target transcripts of non-orthologous genes.

Current approaches address these problems only partially. While hybridization-based techniques fail to address any of the problems (i) to (iii) in a specific manner, they have the benefit of usually allowing the analysis of a large set of transcripts. Sequence-based approaches, so far, offer relatively high stringency and specificity since only perfectly matching probes are retained in the analyses. This usually results in a high loss of genes for subsequent analyses since minor changes in sequences are frequent even among closely related species. Furthermore, the issue of gene orthology has been neglected in the masking approaches, so far.

Orthologous genes are relevant in comparative transcriptomics analyses, because they are derived from a common ancestor. Keeping the focus of the analysis on orthologous genes provides a solid base for biological interpretation of the expression data.

The goal of the 1 mm approach (Methods 1 mm approach) is to mask transcript-unspecific probes and probes matching no transcripts, and to keep only transcript-specific probes that target transcripts of orthologous genes. We permit probes to match transcripts with at most one mismatch in order to account for a possible sequence divergence between the query species and the reference species.

We apply the 1 mm approach, the 0 mm approach described by [4], the gDNA approach described by [7], and the naive approach to *A. thaliana* as reference species and its closely related sister species *A. lyrata* as query species based on the Affymetrix ATH1-121501 microarray designed for *A. thaliana* [8]. The naive approach uses all probes of all probe sets as originally designed by [8].

We require the probe sets of all three masking approaches to contain at least three probes to enhance the reliability of the expression values for a gene as previously proposed by [10].

To assess the performance of the different masking approaches and the non-masking approach, we analyze gene expression data in response to an auxin stimulus for the query species *A. lyrata*, determined by hybridization to an ATH1-121501 microarray.

Auxin is a plant hormone that is involved in virtually all aspects of plant development and is known to induce rapid transcriptome changes as part of its primary signaling response [20], [21].

First, we compare the four approaches with respect to the number of retained probe sets. Second, we perform qRT-PCR experiments for 40 genes, and we compare the four approaches with respect to the Pearson correlation coefficients of the resulting microarray data with the qRT-PCR data. While mismatches can affect hybridization intensities, we show that the tolerance of one mismatch per probe in the proposed approach accurately detects gene expression changes in response to an external treatment of the query species.

### Number and Composition of Probe Sets

**Figure 3:**
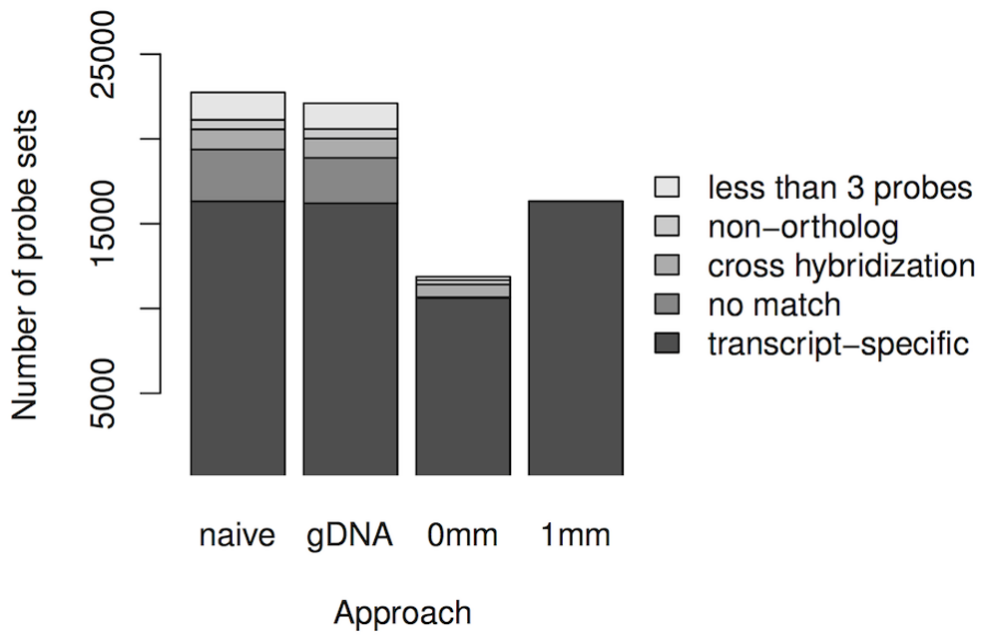
Number of probe sets obtained by the three masking approaches and the naive approach. The height of each bar shows the number of probe sets falling in one of the following categories: *transcript-specific*: retained probe sets targeting orthologs, not affected by cross hybridization, and containing at least 3 probes; *no match*: probe sets matching no transcript in *A. thaliana* or *A. lyrata*; *cross hybridization*: probe sets affected by cross hybridization; *non-ortholog*: probe sets targeting non-orthologs, and *less than 3 probes*: probe sets containing less than 3 matching probes in the 1 mm approach but at least 3 probes in the other approach. The naive approach, the gDNA approach, and the 1 mm approach retain approximately 16000 transcript-specific probe sets, and the 0 mm approach retains approximately 10500 transcript-specific probe sets.

The ATH1-121501 microarray represents approximately 24000 *A. thaliana* genes by 22746 probe sets, which are all contained in the naive approach. 22105 probe sets are retained by the gDNA approach, while 11873 and 16315 probe sets are retained by the 0 mm and the 1 mm approach, respectively (Supplementary Figure S7). Depending on the respective masking approach, retained probe sets can be transcript-specific, transcript-unspecific, can match no transcript or contain less than three probes (Figure 3 and Supplementary Table S1).

First, we consider transcript-specific probe sets, which contain at least three probes that uniquely target transcripts of orthologs as these would represent the genes most relevant in any comparative transcriptomics approach. We find 16315 transcript-specific probe sets retained by the naive approach, 16202 retained by the gDNA approach, 10629 retained by the 0 mm approach, and 16315 retained by the 1 mm approach. The naive, the gDNA, and the 1 mm approach yield approximately the same number of transcript-specific probe sets. These three approaches retain approximately 5500 more transcript-specific probe sets than the 0 mm approach, because the 0 mm approach only retains perfectly matching probes.

Second, we consider transcript-unspecific probe sets, which contain probes that target multiple transcripts or transcripts of non-orthologs. These probe sets would likely result in biased expression values or artifacts and would be undesired in any transcriptomics analysis. Approximately 1700 of the retained probe sets of the naive and the gDNA approach, and approximately 1000 of the 0 mm approach are transcript-unspecific, which comprise approximately 8% of the retained probe sets, respectively. Furthermore, two thirds of the transcript-unspecific probe sets are affected by cross hybridization and one third of the transcript-unspecific probe sets target transcripts of non-orthologous genes in each of the three approaches.

Third, we consider the probe sets that match no transcript in any of the two species with the 1 mm approach. We find that approximately 3000 of the retained probe sets of the naive approach, approximately 2700 of the gDNA approach, and approximately 30 of the 0 mm approach match no transcript. This indicates that approximately 12% of the retained probe sets of the naive and the gDNA approach, and that 0.3% of the retained probe sets of the 0 mm approach match no transcript. In case of the gDNA approach, this may be caused by the possibility that probes target regions on the genomic DNA outside transcribed regions. The 0 mm approach retains only a few probe sets whose probes match no transcript in the 1 mm approach, because in the 0 mm approach probes are checked to be similar to *A. lyrata* but not to *A. thaliana*. Thus, these probes are unspecific for *A. thaliana* and would be uninformative in comparative transcriptomics analysis.

Fourth, we consider those probe sets that contain less than three probes in the 1 mm approach after masking of probes matching no transcripts or multiple transcripts, but contain at least three probes in the other approaches. We find that approximately 1600 of the retained probe sets of the naive and the gDNA approach, and that approximately 200 of the retained probe sets of the 0 mm approach contain at least three probes. This states that approximately 7% of the retained probe sets by the naive and the gDNA approach, and that approximately 2% of the retained probe sets of the 0 mm approach contain at least three probes, whereas they contain less than three probes in the 1 mm approach. Again, for the gDNA approach probes of these probe sets possibly target regions on the genomic DNA outside transcribed regions. And again, these probe sets could result in biased expression values that are undesired in any transcriptomics analysis.

The 1 mm approach efficiently masks probes matching no or multiple transcripts, and probes matching transcripts of non-orthologs. Due to the tolerance of probes with a single mismatch, the number of transcript-specific probe sets retained by the 1 mm approach is similar to that of the gDNA approach and increases from 10629 to 16315 compared to the 0 mm approach (Figure 3 and Supplementary Table S1).

### qRT-PCR Verification

To evaluate the quality of the three masking approaches and the naive approach, we perform qRT-PCR experiments for 40 *A. lyrata* genes (Table 2 and Supplementary Figure S6). We apply the four respective approaches and subsequently compute the Pearson correlation coefficients 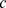 [22] of the auxin induced log-fold changes (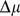) and the 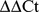 values obtained by qRT-PCR of an independent experiment.

**Table 2:** qRT-PCR verification of masked and non-masked microarray data.

category	naive	gDNA	0 mm	1 mm
(A)	0.91	0.93	0.98	0.98
(B)	0.82		0.95	0.96
(C)	0.83	0.87		0.94
(D)	0.78			0.92

Pearson correlation coefficients of (i) the 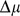 expression responses resulting from the three masking approaches and the naive approach, and (ii) the 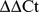 expression responses resulting from qRT-PCR of the genes of category A, B, C, and D (Methods Candidate selection). We find that the 1 mm approach and the 0 mm approach yield similar Pearson correlation coefficients that are higher than those of the gDNA approach and the naive approach.

First, we consider category (A), which contains 20 genes that are present in all three masking approaches and the naive approach. We find Pearson correlation coefficients of 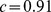 for the naive approach, 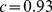 for the gDNA approach, 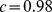 for the 0 mm approach, and 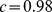 for the 1 mm approach (Table 2 and Supplementary Table S2). Hence, the sequence-based approaches (0 mm and 1 mm ) yield more accurate expression response values than the naive and the gDNA approach for this category. Although the 1 mm approach permits single mismatches and the more stringent 0 mm approach does not, both approaches yield similarly high Pearson correlation coefficients.

Second, we consider category (B), which contains 22 genes that are present in the naive, the 0 mm, and the 1 mm approach, and we find Pearson correlation coefficients of 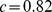 for the naive approach, 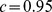 for the 0 mm approach, and 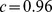 for the 1 mm approach. Again, both sequence-based approaches yield similar, but higher Pearson correlation coefficients than the naive approach.

The similar Pearson correlation coefficients indicate that, despite probes matching with one mismatch can have a reduced hybridization efficacy (Supplementary Figures S1 and S2) [1], [23], [24], the accuracy of the log-fold changes (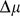) is not reduced by using probes matching with a single mismatch (Supplementary Figure S3). To account for the reduced hybridization efficacy of probes matching with one mismatch, we suggest a correction approach using a fourth-degree polynomial, which corrects the nominal expression values according to the positional effect of the respective mismatch but does not have a significant effect on the log-fold changes (Supplementary Figures S4 and S5).

Third, we consider category (C), which contains 36 genes that are present in the naive, the gDNA, and the 1 mm approach, and we find Pearson correlation coefficients of 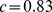 for the naive approach, 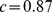 for the gDNA approach, and 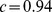 for the 1 mm approach, stating that also for the genes of category (C) the 1 mm approach yields higher Pearson correlation coefficients than the naive and the gDNA approach. This difference might be explained by the fact that, even though the 1 mm and the gDNA approaches retain the probe sets of the 36 genes of category (C), the probe sets contain different probes. The probe sets of the gDNA approach lack approximately 30% of the probes matching with at most one mismatch, but approximately 35% of probes possibly match regions on the DNA outside transcribed regions, or match multiple targets (Table 1).

Fourth, we consider category (D), which contains 40 genes that are present in the naive and the 1 mm approach, and we find Pearson correlation coefficients of 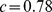 for the naive approach and 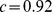 for the 1 mm approach, stating that also for genes of category (D) the 1 mm approach yields a higher Pearson correlation coefficient than the naive approach.

For all four categories, we find similar results for Spearman and Kendall correlation as for the Pearson correlation (Supplementary Table S2).

In summary, we find that both sequence-based approaches yield more accurate expression responses than the naive and the gDNA approach. This finding is interesting, because the 1 mm approach retains approximately 5500 additional transcript-specific probe sets than the more stringent 0 mm approach, which allows a more comprehensive yet still accurate analysis of transcriptome changes/responses.

## Conclusions

We address the problem of obtaining reliable expression response data for microarray-based comparative transcriptomics studies of a reference species and a closely related query species. We propose an approach that can be used if whole-transcriptome sequence information is available for the query species and that addresses the problems of (i) probes targeting no transcript, (ii) probes affected by cross hybridization, and (iii) probes targeting transcripts of non-orthologous genes.

We find that the 1 mm and the 0 mm approach yield a similar accuracy in qRT-PCR verification of the expression response values and outperform the naive and the gDNA approach, indicating that imperfectly matching probes with a single mismatch do not reduce the quality of the recorded 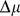 log fold-changes.

However, using imperfectly matching probes with a single mismatch increases the number of transcript-specific probes per probe set and the number of transcript-specific probe sets of orthologous genes from 10629 for the 0 mm approach to 16315 for the 1 mm approach. In addition, the 1 mm approach reduces the number of probe sets that are potentially affected by cross hybridization or that target transcripts of non-orthologous genes, and we conjecture that the proposed 1 mm approach will considerably improve future comparative transcriptomics studies.

## Supporting Information

Figure S1 **Number of probes of the 1 mm mask that match the transcripts of** ***A. lyrata*** **without any mismatch (none) or with one mismatch at a specific position.** The number of probes with a mismatch is similar for all mismatch positions.
(TIFF)

Figure S2

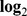
 **expression values of probes of the 1 mm mask that match the transcripts of** ***A. lyrata*** **without any mismatch (none) or with one mismatch at a specific position.** The expression values measured depend on the occurrence of a mismatch and its position within the probe sequence. Hence, a correction for this positional bias would be required to compare expression values between *A. thaliana* and *A.* *lyrata*.(TIFF)

Figure S3

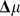
 **expression responses of probes of the 1 mm mask that match the transcripts of** ***A. lyrata*** **without any mismatch (none) or with one mismatch at a specific position.** The expression responses of probes are similar for all mismatch position as well as for the perfectly matching probes. In contrast to the 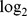 expression values, the comparison of 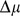 expression responses between *A. thaliana* and *A. lyrata* does not require a correction.(TIFF)

Figure S4

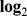
 **expression values of probes of the 1 mm mask.** (A) 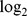 expression values of probes of the 1 mm mask that match the transcripts of *A. thaliana* and *A. lyrata* without any mismatch (none) or with one mismatch at a specific position. The expression values measured depend on the occurrence of a mismatch and its position within the probe sequence. To correct for this positional bias we fit a fourth-degree polynomial to the data (red curve). (B) Corrected 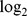 expression values based on the polynomial fit. The corrected expression values are not affected by the occurrence of a mismatch and its position within the probe sequence any more.(TIFF)

Figure S5

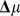
 **expression responses of probes of the 1** **mm mask.** (A) 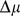 expression responses of probes of the 1 mm mask that match the transcripts of *A. thaliana* and *A. lyrata* without any mismatch (none) or with one mismatch at a specific position. The expression responses of probes are similar for all mismatch position as well as for the perfectly matching probes. (B) Corrected (Figure S4) 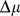 expression responses of probes of the 1 mm mask. The expression responses of probes are similar to the uncorrected expression responses in (A). The suggested correction does not have a significant effect on the expression responses.(TIFF)

Figure S6
**Scatterplots and Pearson correlation coefficients.** Correlation coefficients of (i) the 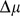 expression responses of *A. lyrata* resulting from the three masking approaches and the naive approach, and (ii) the 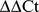 expression responses resulting from qRT-PCR of the genes of category A, B, C, and D (Methods Candidate selection).(TIFF)

Figure S7 **Frequency of probes per probe set.** The height of the bars represents the absolute frequency of probe sets containing a defined number of probes. For each number of probes three bars are shown, one for each of the three probe masking approaches. Total number of probe sets: 16315 (1 mm approach), 11873 (0 mm approach), and 22105 (gDNA approach).
(TIFF)

Table S1 **Number of probe sets and probes, and average number of probes per probe set for all three masking approaches and the naive approach.** Values are rounded to the first or second position after decimal point. Additionally, the number of probe sets falling in one of the following categories are listed: *transcript-specific*: probe sets targeting orthologs, not affected by cross hybridization, and containing at least 3 probes; *transcript-unspecific* probe sets that can be separated in *cross hybridization*: probe sets affected by cross hybridization and *non-ortholog*: probe sets targeting non-orthologs; *no match*: probe sets matching no transcript in *A. thaliana* or *A. lyrata*; and *less than 3 probes*: probe sets containing less than 3 matching probes in the 1 mm approach, but at least 3 probes in the other approach. The 1 mm approach retains a similar number of transcript-specific probe sets as the gDNA and the naive approach, but retains more transcript-specific probe sets than the 0 mm approach.
(PDF)

Table S2
**Pearson, Spearman and Kendall correlation coefficients.** Correlation coefficients of (i) the 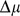 expression values resulting from the three masking approaches and the naive approach, and (ii) the 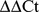 expression values resulting from qRT-PCR of the genes of category A, B, C, and D (Methods Candidate selection). The 1 mm approach and the 0 mm approaches yield similar correlation coefficients that are higher than those of the gDNA and the naive approaches.(PDF)

Table S3 **Primer sequences for** ***A. lyrata*** **of the 40 candidate genes used for verification by qRT-PCR.** The locus identifier for *A. thaliana* is given by the TAIR id and that for *A. lyrata* by the Phytozome gene id.
(PDF)

Table S4 **Probe sets of the 40 candidate genes containing the position of the mismatch.** A mismatch can occur at position 1 to 25. A “0” indicates that the probe matches perfectly without any mismatch and a “–” Indicates that the probe is masked. Originally, each of the 40 probe sets consists of 11 probes.
(PDF)
