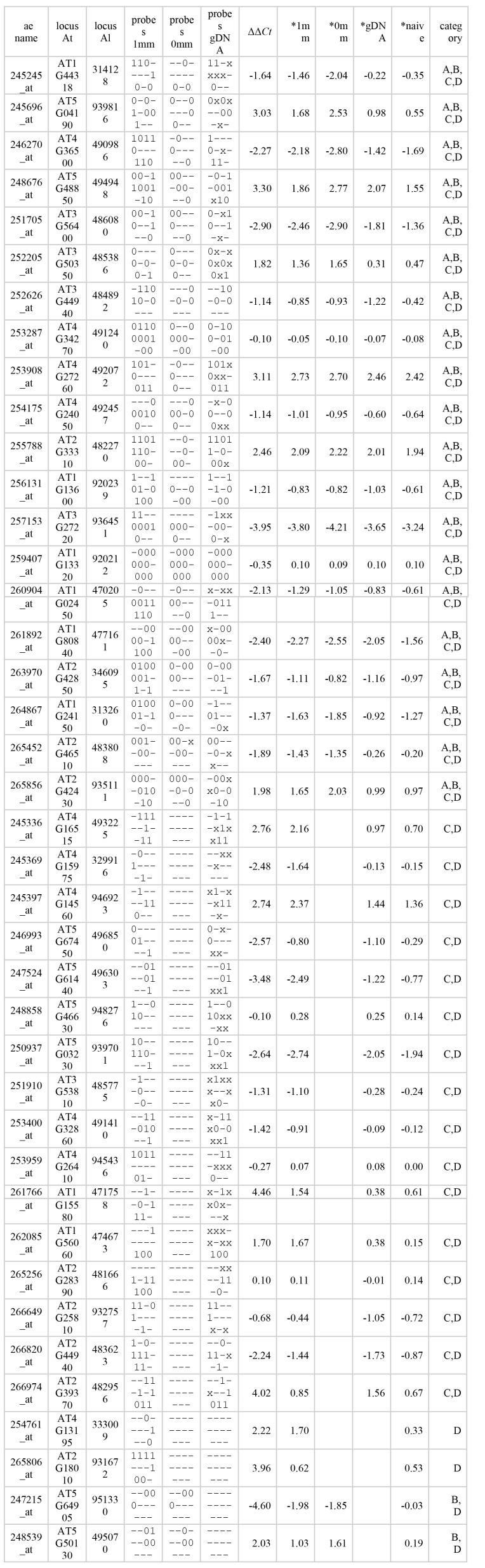# Correction: Optimized Probe Masking for Comparative Transcriptomics of Closely Related Species

**DOI:** 10.1371/annotation/a5a47872-2b0d-42bb-b24c-e312bb417e5e

**Published:** 2014-01-17

**Authors:** Yvonne Poeschl, Carolin Delker, Jana Trenner, Kristian Karsten Ullrich, Marcel Quint, Ivo Grosse

The content of columns four and five are incorrect in Table 1. Please see the corrected Table 1 here: 

**Figure:**