# Supplementary figures and images for: Optimized Probe Masking for Comparative Transcriptomics of Closely Related Species
Source: PLoS One. 2013 Nov 8;8(11):e78497. doi: 10.1371/journal.pone.0078497 (PMC3832635; doi:10.1371/journal.pone.0078497)

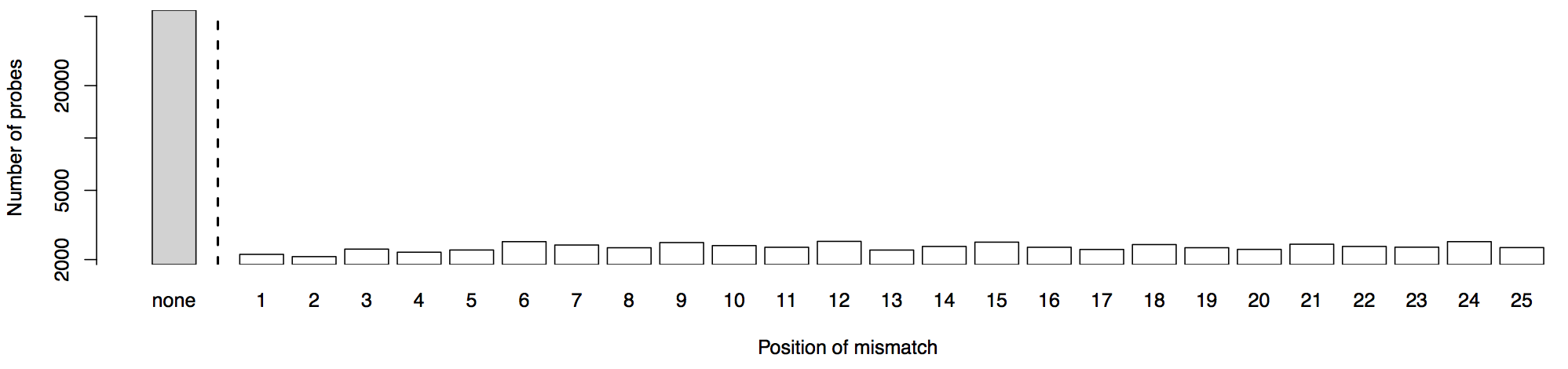

Supplement: Figure S1 — Number of probes of the 1 mm mask that match the transcripts of A. lyrata without any mismatch (none) or with one mismatch at a specific position. The number of probes with a mismatch is similar for all mismatch positions. (TIFF) [file pone.0078497.s001.tiff]

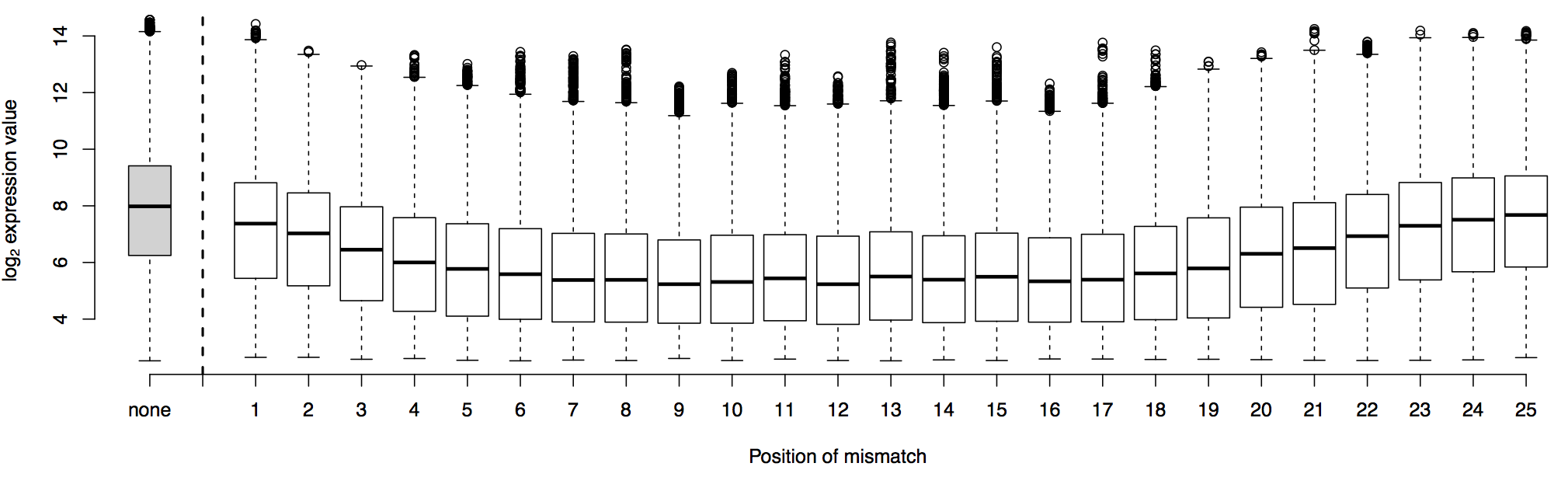

Supplement: Figure S2 — expression values of probes of the 1 mm mask that match the transcripts of A. lyrata without any mismatch (none) or with one mismatch at a specific position. The expression values measured depend on the occurrence of a mismatch and its position within the probe sequence. Hence, a correction for this positional bias would be required to compare expression values between A. thaliana and A. lyrata. (TIFF) [file pone.0078497.s002.tiff]

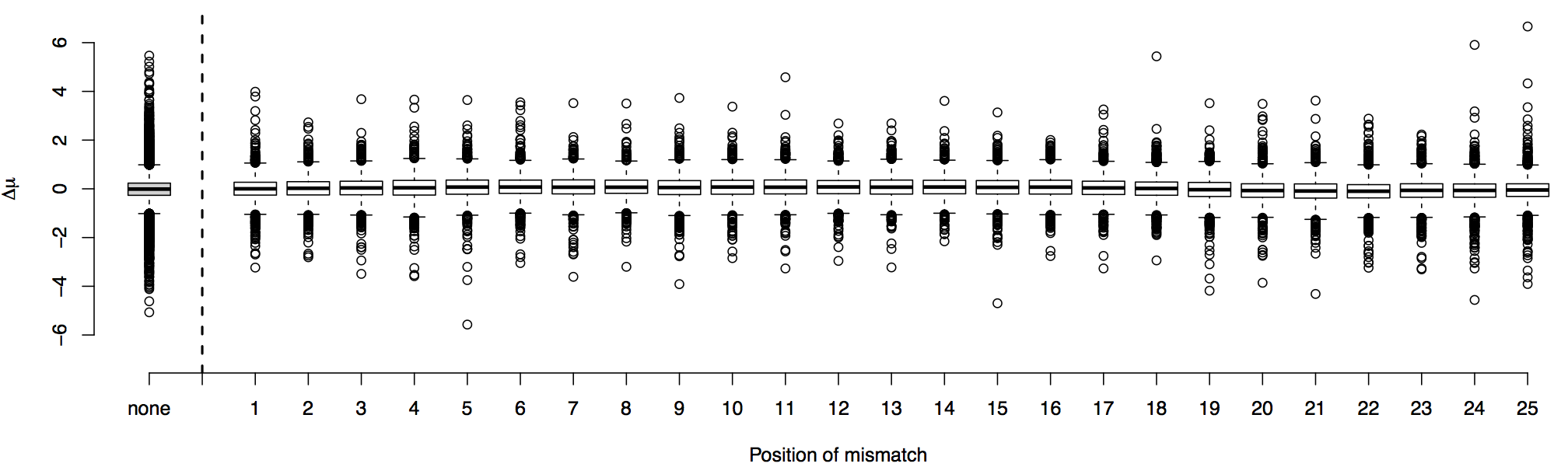

Supplement: Figure S3 — expression responses of probes of the 1 mm mask that match the transcripts of A. lyrata without any mismatch (none) or with one mismatch at a specific position. The expression responses of probes are similar for all mismatch position as well as for the perfectly matching probes. In contrast to the expression values, the comparison of expression responses between A. thaliana and A. lyrata does not require a correction. (TIFF) [file pone.0078497.s003.tiff]

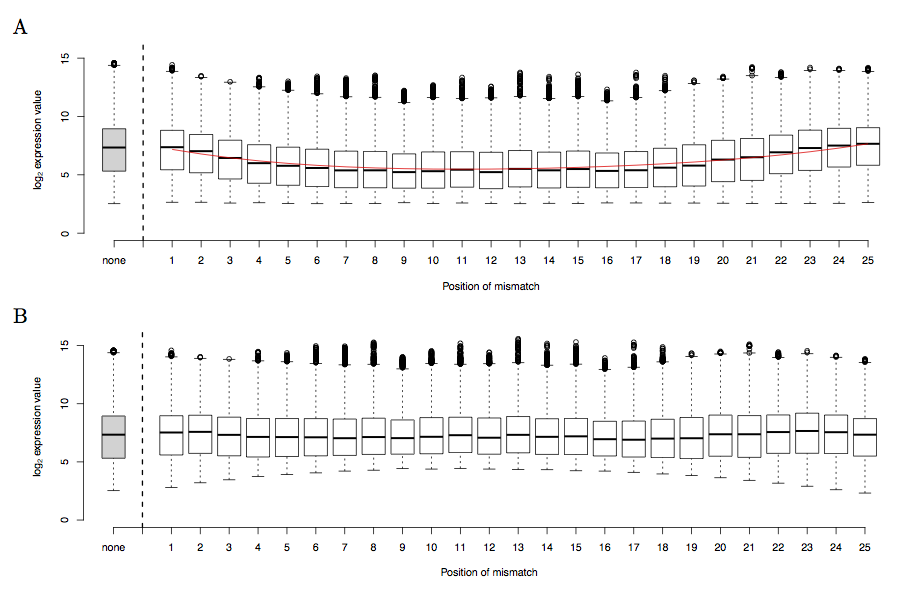

Supplement: Figure S4 — expression values of probes of the 1 mm mask. (A) expression values of probes of the 1 mm mask that match the transcripts of A. thaliana and A. lyrata without any mismatch (none) or with one mismatch at a specific position. The expression values measured depend on the occurrence of a mismatch and its position within the probe sequence. To correct for this positional bias we fit a fourth-degree polynomial to the data (red curve). (B) Corrected expression values based on the polynomial fit. The corrected expression values are not affected by the occurrence of a mismatch and its position within the probe sequence any more. (TIFF) [file pone.0078497.s004.tiff]

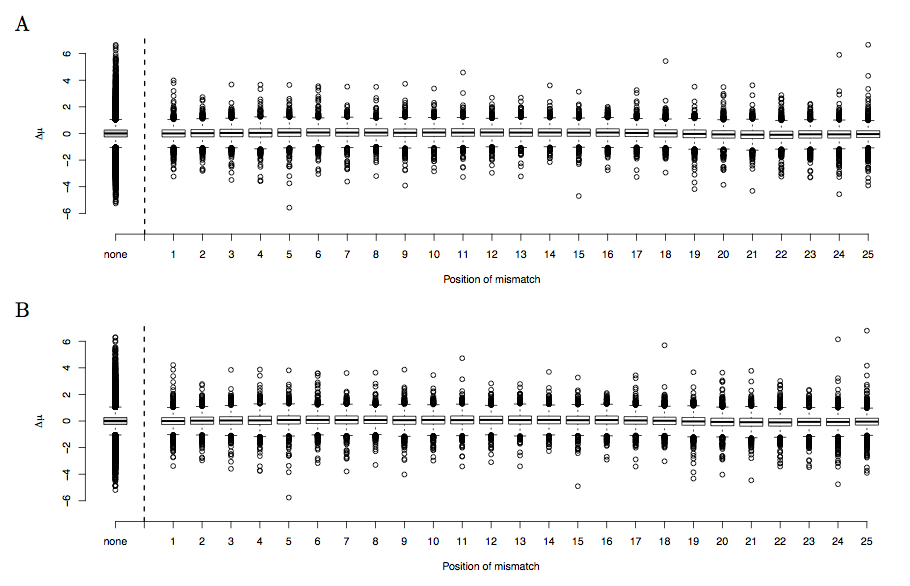

Supplement: Figure S5 — expression responses of probes of the 1 mm mask. (A) expression responses of probes of the 1 mm mask that match the transcripts of A. thaliana and A. lyrata without any mismatch (none) or with one mismatch at a specific position. The expression responses of probes are similar for all mismatch position as well as for the perfectly matching probes. (B) Corrected (Figure S4) expression responses of probes of the 1 mm mask. The expression responses of probes are similar to the uncorrected expression responses in (A). The suggested correction does not have a significant effect on the expression responses. (TIFF) [file pone.0078497.s005.tiff]

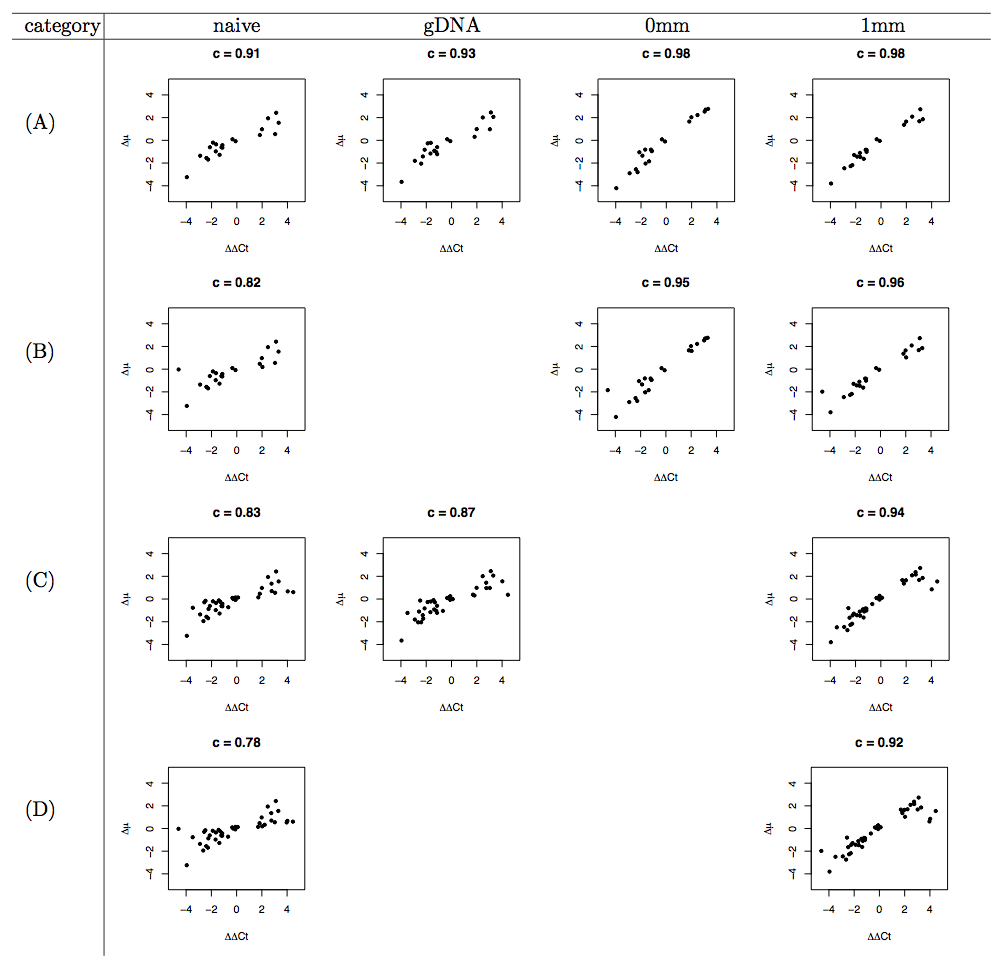

Supplement: Figure S6 — Scatterplots and Pearson correlation coefficients. Correlation coefficients of (i) the expression responses of A. lyrata resulting from the three masking approaches and the naive approach, and (ii) the expression responses resulting from qRT-PCR of the genes of category A, B, C, and D (Methods Candidate selection). (TIFF) [file pone.0078497.s006.tiff]

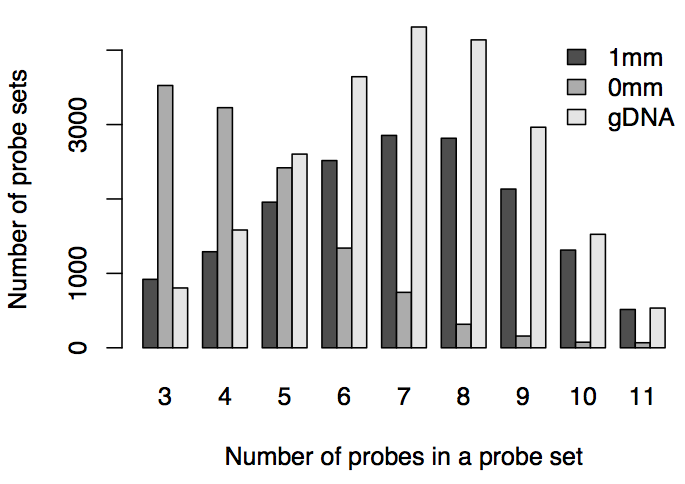

Supplement: Figure S7 — Frequency of probes per probe set. The height of the bars represents the absolute frequency of probe sets containing a defined number of probes. For each number of probes three bars are shown, one for each of the three probe masking approaches. Total number of probe sets: 16315 (1 mm approach), 11873 (0 mm approach), and 22105 (gDNA approach). (TIFF) [file pone.0078497.s007.tiff]
