# Supplementary material for: Optimized Probe Masking for Comparative Transcriptomics of Closely Related Species
Source: PLoS One. 2013 Nov 8;8(11):e78497. doi: 10.1371/journal.pone.0078497 (PMC3832635; doi:10.1371/journal.pone.0078497)
Supplement: Table S1 — Number of probe sets and probes, and average number of probes per probe set for all three masking approaches and the naive approach. Values are rounded to the first or second position after decimal point. Additionally, the number of probe sets falling in one of the following categories are listed: transcript-specific: probe sets targeting orthologs, not affected by cross hybridization, and containing at least 3 probes; transcript-unspecific probe sets that can be separated in cross hybridization: probe sets affected by cross hybridization and non-ortholog: probe sets targeting non-orthologs; no match: probe sets matching no transcript in A. thaliana or A. lyrata; and less than 3 probes: probe sets containing less than 3 matching probes in the 1 mm approach, but at least 3 probes in the other approach. The 1 mm approach retains a similar number of transcript-specific probe sets as the gDNA and the naive approach, but retains more transcript-specific probe sets than the 0 mm approach. (PDF) [file pone.0078497.s008.pdf]

Table S1. Number of probe sets and probes, and average number of probes per probe set for all three masking approaches and the naive approach. Values are rounded to the first or second position after decimal point. Additionally, the number of probe sets falling in one of the following categories are listed: *transcript-specific*: probe sets targeting orthologs, not affected by cross hybridization, and containing at least 3 probes; *transcript-unspecific* probe sets that can be separated in *cross hybridization*: probe sets affected by cross hybridization and *non-ortholog*: probe sets targeting non-orthologs; *no match*: probe sets matching no transcript in *A. thaliana* or *A. lyrata*; and *less than 3 probes*: probe sets containing less than 3 matching probes in the 1mm approach, but at least 3 probes in the other approach. The 1mm approach retains a similar number of transcript-specific probe sets as the gDNA and the naive approach, but retains more transcript-specific probe sets than the 0mm approach.

	**naive**	**gDNA**	**0mm**	**1mm**
transcript-specific	16315 (71.7 %)	16202 (73.3 %)	10629 (89.5 %)	16315 (100 %)
transcript-unspecific:	1749 (7.7 %)	1701 (7.7 %)	1012 (8.5 %)	0
cross hybridization	1183	1149	749	0
non-ortholog	566	552	263	0
no match	3067 (13.5 %)	2682 (12.1 %)	33* (0.3 %)	0
less than 3 probes	1615 (7.1 %)	1520 (6.9 %)	199 (1.7 %)	0
total number of probe sets	22746	22105	11873	16315
total number of probes	250103	154698	54281	113303
average number of probes per probe set	11.00	7.00	4.57	6.95

*The 0mm approach uses the targets for *A. thaliana* annotated by Affymetrix to determine the target sequences of *A. lyrata*, by aligning the transcript sequences of *A. lyrata* to the annotated target sequences of *A. thaliana*. The sequences of the probes are aligned to the sequences of target transcripts of *A. lyrata*. Only probes are retained that perfectly match the target transcripts of *A. lyrata*.

The 1mm approach aligns the sequences of the probes to the sequences of protein-coding transcripts of *A. thaliana* and *A. lyrata*. A probe set has *no match* if the probes do not match any transcript in *A. thaliana* or *A. lyrata*.
