# Supplementary material for: Optimized Probe Masking for Comparative Transcriptomics of Closely Related Species
Source: PLoS One. 2013 Nov 8;8(11):e78497. doi: 10.1371/journal.pone.0078497 (PMC3832635; doi:10.1371/journal.pone.0078497)
Supplement: Table S2 — Pearson, Spearman and Kendall correlation coefficients. Correlation coefficients of (i) the expression values resulting from the three masking approaches and the naive approach, and (ii) the expression values resulting from qRT-PCR of the genes of category A, B, C, and D (Methods Candidate selection). The 1 mm approach and the 0 mm approaches yield similar correlation coefficients that are higher than those of the gDNA and the naive approaches. (PDF) [file pone.0078497.s009.pdf]

Table S2. Pearson, Spearman and Kendall correlation coefficients of the $\Delta\mu$ expression values resulting from the three masking approaches and the naive approach, and the $\Delta\Delta$Ct expression values resulting from qRT-PCR of the genes of category A, B, C, and D (Methods Candidate selection). The 1mm and the 0mm approaches yield similar correlation coefficients, but larger ones than that for the gDNA and the naive approaches.

category	mask	Pearson	Spearman	Kendall
A	1mm	0.980	0.961	0.853
	gDNA	0.929	0.902	0.758
	0mm	0.978	0.950	0.853
	naive	0.913	0.905	0.747
B	1mm	0.961	0.956	0.827
	0mm	0.952	0.939	0.818
	naive	0.818	0.820	0.662
C	1mm	0.938	0.931	0.797
	gDNA	0.867	0.898	0.717
	naive	0.830	0.877	0.692
D	1mm	0.920	0.920	0.774
	naive	0.777	0.856	0.669
