# Supplementary material for: Optimized Probe Masking for Comparative Transcriptomics of Closely Related Species
Source: PLoS One. 2013 Nov 8;8(11):e78497. doi: 10.1371/journal.pone.0078497 (PMC3832635; doi:10.1371/journal.pone.0078497)
Supplement: Table S3 — Primer sequences for A. lyrata of the 40 candidate genes used for verification by qRT-PCR. The locus identifier for A. thaliana is given by the TAIR id and that for A. lyrata by the Phytozome gene id. (PDF) [file pone.0078497.s010.pdf]

**Table S3.** Primer sequences of the 40 candidate genes used for verification by qRT-PCR. The locus identifier for *A. thaliana* is given by the TAIR id and for *A. lyrata* by the Phytozome gene id.

| ae name | locus At | locus Al | forward primer | reverse primer |
|---|---|---|---|---|
| 245245_at | AT1G44318 | 314128 | AACTGGGCACGGTGGGATCG | CGCGCCTACACGACCATCCA |
| 245336_at | AT4G16515 | 493225 | GCTGCCGCTCGTCGGTTAGG | CGACCCCACCCAACACTCGC |
| 245369_at | AT4G15975 | 329916 | TAGCCGCTTCAACCGCACCG | GGTTGCGGCGGAGAACGTGA |
| 245397_at | AT4G14560 | 946923 | ACCGAGCTTCGTTTGGGATTACCTG | GGAGGCCATCCCACGATTTGTGTT |
| 245696_at | AT5G04190 | 939816 | GCTCGTCCATGGGCTCCACC | CCGGCTCGGCGGTCATAACG |
| 246270_at | AT4G36500 | 490986 | GGTGCTGGTGGTGTTCCGACC | CGGGTGGCTAAATTTGCCTGTTGG |
| 246993_at | AT5G67450 | 496850 | ACGGAAGTAGCAGCAACAGCGT | GGCCACCAATAGCACTTTCTTCCGA |
| 247215_at | AT5G64905 | 951330 | GGCGATTTTTCGTCATCTCACAGCG | GTCTTGGTCTTCCCTCGCGCTT |
| 247524_at | AT5G61440 | 496303 | ACGATGCAGCCTCGGGCCA | TTCCCCAACCGATGCCAAAGCC |
| 248539_at | AT5G50130 | 495070 | GCCAGGGCGCAGCTACAACA | TGGGTGCATAGCTTGAAAGCCACA |
| 248676_at | AT5G48850 | 494948 | ACCCACCAAGACCGCTCGCT | TGTATACCGCCTCTGCCGACAAGT |
| 248858_at | AT5G46630 | 948276 | CGAAGATGCCGGTGGCTGCT | CGACGTCATCACGGTAGGTGCG |
| 250937_at | AT5G03230 | 939701 | CGCACGAGTATTTAGCGCGGC | TTCGCCGTTCCACCGATTCCTTC |
| 251705_at | AT3G56400 | 486080 | GGGTGCAAGGCAACAAAGCAAGT | TGCGTTGGTGTTACACGTGTGGT |
| 251910_at | AT3G53810 | 485775 | GGCCGGGACGGTTTTAGCGG | ATCAAACATCACCTCCCACCGGAGA |
| 252205_at | AT3G50350 | 485386 | CCGGGGGTAGTGCGTCGTCT | GCTCTTTCCACGGCGGCGAT |
| 252626_at | AT3G44940 | 484892 | CGGTCCTCTGCCTCAATGGCG | AGTGGTGGGATGGTGACAGGAGG |
| 253287_at | AT4G34270 | 491240 | GTGAAAACTGTTGGAGAGAAGCAA | TCAACTGGATACCCTTTCGCA |
| 253400_at | AT4G32860 | 491410 | AGCGCCAACGCCAAGCGTAT | ACGTCGGGAAACACTGCCGC |
| 253908_at | AT4G27260 | 492072 | TGGATGGAAACACGCCGATCCC | AAGCGGGCCTATGGACTTGTCACT |
| 253959_at | AT4G26410 | 945436 | ACTGCTGCTGATGCGACGGTG | GAGCAGAGCGCATGGCGGAA |
| 254175_at | AT4G24050 | 492457 | ACCGTCCCTCAAGCAGCAGCA | TTGGATCCGAGTCCAGACGGCG |
| 254761_at | AT4G13195 | 333009 | ACTCACACTAGACGGAGTCGCAGT | TGAGCTTCAGCTCCGAACTCTCTCC |
| 255788_at | AT2G33310 | 482270 | ACCAAGCTACGAAATCTGCGAGGG | ACCCAACTGGCACTTTCCCATTCAC |
| 256131_at | AT1G13600 | 920239 | GGGAATCTGCTCGTAGGTCA | TCAGATACGCGGTTCAGCTT |
| 257153_at | AT3G27220 | 936451 | GGGAGGCTTCATGTGATGGGTGG | GCCCTGTGTGGTCCACCTCG |
| 259407_at | AT1G13320 | 920212 | AGACAAGGTTCACTCAATCCGTG | CATTCAGGACCAAACTCTTCAGC |
| 260904_at | AT1G02450 | 470205 | TAGCGGCGGAGAAAGATCCGGT | GCGGCTTCAAATCCGTACGACACT |
| 261766_at | AT1G15580 | 471758 | GGCCTCTCCGGAAGTGGAGAGTAA | AACCGGTGGCCAACCCCACAA |
| 261892_at | AT1G80840 | 477161 | AGGACCAGTCCGTGTTGGTTGC | GCTGCTGCGGGTGTTGAAGC |
| 262085_at | AT1G56060 | 474673 | CGTATGTGACAGCTCCGCCACC | AGCAGCAGCACATTGCAGCCA |
| 263970_at | AT2G42850 | 346095 | AGGAGGGCGCTGAGAAGCCA | TGGCCATGGCGTAAGAGGTTGTG |
| 264867_at | AT1G24150 | 313260 | TCAGAGGGGAAGCGATGTGTGCT | TCGAACTGCTGCTGCTACGGC |
| 265256_at | AT2G28390 | 481666 | AACTCTATGCAGCATTTGATCCACT | TGATTGCATATCTTTATCGCCATC |
| 265452_at | AT2G46510 | 483808 | GGCGGTCCGGGGAGGTGTTA | TCGTTCTGATTCCCGCAGATTTCGC |
| 265806_at | AT2G18010 | 931672 | CCGTTTACGTGGGACCGAACCG | CCTCGGCTAGTCGGAGCAACG |
| 265856_at | AT2G42430 | 935111 | GCTGTCGTCACCATCGCCTACG | GGCCGGCGATCTGTGCCTTC |
| 266649_at | AT2G25810 | 932757 | CGCCATGGCCACCGACAGTT | GTGACCGCGGGGTTGAGGTG |
| 266820_at | AT2G44940 | 483623 | CGGCGAGCTTCCTCGTCCAG | GCTCGACTCGGCTCGGCTTC |
| 266974_at | AT2G39370 | 482956 | CATGCGCGTCTTCCGCTGC | ACGATCTCCTATGGCTCCCGGAAA |

ae: array element, At: *Arabidopsis thaliana*, Al: *Arabidopsis lyrata*
