# Supplementary material for: Optimized Probe Masking for Comparative Transcriptomics of Closely Related Species
Source: PLoS One. 2013 Nov 8;8(11):e78497. doi: 10.1371/journal.pone.0078497 (PMC3832635; doi:10.1371/journal.pone.0078497)
Supplement: Table S4 — Probe sets of the 40 candidate genes containing the position of the mismatch. A mismatch can occur at position 1 to 25. A “0” indicates that the probe matches perfectly without any mismatch and a “–” Indicates that the probe is masked. Originally, each of the 40 probe sets consists of 11 probes. (PDF) [file pone.0078497.s011.pdf]

Table S4. Probe sets of the 40 candidate genes containing the position of the mismatch. A mismatch can occur at position 1 to 25. A "0" indicates that the probe matches perfectly without any mismatch and a "–" indicates that the probe is masked. Each of the 40 probe sets originally consists of 11 probes.

ae name	probes matching transcripts of At											probes matching transcripts of Al										
	**1**	**2**	**3**	**4**	**5**	**6**	**7**	**8**	**9**	**10**	**11**	**1**	**2**	**3**	**4**	**5**	**6**	**7**	**8**	**9**	**10**	**11**
245245_at	0	0	0	–	–	–	–	0	0	–	0	10	9	0	–	–	–	–	24	0	–	0
245696_at	0	–	0	–	0	–	0	0	0	–	–	0	–	0	–	6	–	0	0	3	–	–
246270_at	0	0	0	0	0	–	–	–	0	0	0	25	0	1	14	0	–	–	–	9	10	0
248676_at	0	0	–	0	0	0	0	0	–	0	0	0	0	–	25	4	0	0	17	–	8	0
251705_at	0	0	–	0	0	–	–	0	–	–	0	0	0	–	22	0	–	–	1	–	–	0
252205_at	0	–	–	–	0	–	0	–	0	–	0	0	–	–	–	0	–	0	–	0	–	24
252626_at	–	0	0	0	0	0	–	0	–	–	–	–	13	13	0	23	0	–	0	–	–	–
253287_at	0	0	0	0	0	0	0	0	–	0	0	0	15	10	0	0	0	0	8	–	0	0
253908_at	0	0	0	–	0	–	–	–	0	0	0	8	0	9	–	0	–	–	–	0	6	14
254175_at	–	–	–	0	0	0	0	0	0	–	–	–	–	–	0	0	0	4	0	0	–	–
255788_at	0	0	0	0	0	0	0	–	0	0	–	3	17	0	18	1	17	0	–	0	0	–
256131_at	0	–	–	0	0	0	–	0	0	0	0	24	–	–	22	0	20	–	0	18	0	0
257153_at	0	0	–	–	0	0	0	0	0	–	–	11	15	–	–	0	0	0	12	0	–	–
259407_at	–	0	0	0	0	0	0	–	0	0	0	–	0	0	0	0	0	0	–	0	0	0
260904_at	–	0	–	–	0	0	0	0	0	0	0	–	0	–	–	0	0	21	10	17	5	0
261892_at	–	–	0	0	0	0	–	0	0	0	0	–	–	0	0	0	0	–	15	15	0	0
263970_at	0	0	0	0	0	0	0	–	0	–	0	0	14	0	0	0	0	25	–	3	–	9
264867_at	0	0	0	0	0	0	–	0	–	0	–	0	8	0	0	0	12	–	12	–	0	–
265452_at	0	0	0	–	–	0	0	–	–	–	–	0	0	12	–	–	0	0	–	–	–	–
265856_at	0	0	0	–	–	0	0	0	–	0	0	0	0	0	–	–	0	15	0	–	6	0
245336_at	–	0	0	0	–	–	0	–	–	0	0	–	17	3	20	–	–	23	–	–	1	8
245369_at	–	0	–	–	16	–	–	–	–	0	–	–	0	–	–	24	–	–	–	–	8	–
245397_at	–	0	–	–	–	–	0	0	0	–	–	–	9	–	–	–	–	10	22	0	–	–
246993_at	0	–	–	–	0	0	–	–	–	–	0	0	–	–	–	0	15	–	–	–	–	14
247524_at	–	–	0	0	–	–	0	0	–	–	0	–	–	0	16	–	–	0	23	–	–	19
248858_at	0	–	–	0	0	0	–	–	–	–	–	8	–	–	0	12	0	–	–	–	–	–
250937_at	0	0	–	–	0	0	0	–	–	–	0	7	0	–	–	25	11	0	–	–	–	19
251910_at	–	0	–	–	–	0	–	–	–	0	–	–	5	–	–	–	0	–	–	–	0	–
253400_at	–	–	0	0	–	0	0	0	–	–	0	–	–	21	22	–	0	10	0	–	–	5
253959_at	0	0	0	0	–	–	–	–	0	0	–	13	0	22	9	–	–	–	–	0	17	–
261766_at	–	–	0	–	–	0	–	0	0	0	–	–	–	21	–	–	0	–	15	11	18	–
262085_at	–	–	–	0	–	–	–	–	0	0	0	–	–	–	7	–	–	–	–	2	0	0
265256_at	–	–	–	–	0	–	0	0	0	0	0	–	–	–	–	11	–	6	7	23	0	0
266649_at	0	0	–	0	0	–	–	–	–	0	–	18	16	–	0	24	–	–	–	–	9	–
266820_at	0	–	0	–	0	0	0	–	0	0	–	19	–	0	–	17	23	22	–	17	22	–
266974_at	–	–	0	0	–	0	–	0	0	0	0	–	–	13	13	–	14	–	20	0	7	4
254761_at	–	–	0	–	–	–	–	0	–	–	0	–	–	0	–	–	–	–	10	–	–	0
265806_at	0	0	0	0	–	–	–	0	0	0	–	14	18	1	25	–	–	–	9	0	0	–
247215_at	–	–	0	0	0	–	–	–	–	–	–	–	–	0	0	0	–	–	–	–	–	–
248539_at	–	–	0	0	–	–	0	0	–	–	–	–	–	0	7	–	–	0	0	–	–	–

ae: array element, At: *Arabidopsis thaliana*, Al: *Arabidopsis lyrata*
